# Using Misoprostol for Primary versus Secondary Prevention of Postpartum Haemorrhage – Do Costs Matter?

**DOI:** 10.1371/journal.pone.0164718

**Published:** 2016-10-18

**Authors:** Susmita Chatterjee, Anupam Sarkar, Krishna D. Rao

**Affiliations:** 1 Public Health Foundation of India, New Delhi, India; 2 Johns Hopkins University, Baltimore, United States of America; Stellenbosch University, SOUTH AFRICA

## Abstract

**Background:**

Postpartum heammorrhage (PPH), defined as blood loss greater than or equal to 500 ml within 24 hours after birth, is the leading cause of maternal deaths globally and in India. Misoprostol is an important option for PPH management in setting where oxytocin (the gold standard for PPH prevention and treatment) in not available or not feasible to use. For the substantial number of deliveries which take place at home or at lower level heatlh facilities in India, misoprostol pills can be adminstered to prevent PPH. The standard approach using misoprostol is to administer it prophylactically as primary prevention (600 mcg). An alternative strategy could be to administer misoprostol only to those who are at high risk of having PPH i.e. as secondary prevention.

**Methods:**

This study reports on the relative cost per person of a strategy involving primary versus secondary prevention of PPH using misoprostol. It is based on a randomized cluster trial that was conducted in Bijapur district in Karnataka, India between December 2011 and March 2014 among pregnant women to compare two community-level strategies for the prevention of PPH: primary and secondary. The analysis was conducted from the government perspective using an ingredient approach.

**Results:**

The cluster trial showed that there were no significant differences in clinical outcomes between the two study arms. However, the results of the cost analysis show that there is a difference of INR 6 (US$ 0.1) per birth for implementing the strategies primary versus secondary prevention. In India where 14.9 million births take place at sub-centres and at home, this additional cost of INR 6 per birth translates to an additional cost of INR 94 (US$ 1.6) million to the government to implement the primary prevention compared to the secondary prevention strategy.

**Conclusion:**

As clinical outcomes did not differ significantly between the two arms in the trial, taking into account the difference in costs and potential issues with sustainability, secondary prevention might be a more strategic option.

## Introduction

Postpartum Haemorrhage (PPH) is commonly defined as a blood loss of 500 ml or more within 24 hours after birth. PPH is the leading cause of maternal mortality in low and middle-income countries and the primary cause of nearly one quarter of all maternal deaths globally [1]. Oxytocin (10 international units administered intravenously or intramuscularly) is the recommended uterotonic drug for the prevention of PPH [1]. However, in settings where oxytocin is unavailable, the use of oral misoprostol (600 mcg) is recommended.

PPH is the leading cause of maternal deaths in India as well. One study reported that the proportion of maternal deaths caused by haemorrhage was 24.5 percent during 2001–03 in India [2]. Following WHO guidelines, the government’s policy for preventing PPH in India is to administer 10 units of oxytocin intramuscularly immediately after delivery as a component of active management of third stage of labour. However, as oxytocin requires refrigeration and needs to be administered by a trained health professional, it is available only at primary health centres (PHC) or at higher level health facilities. Misoprostol is stable at room temperature and is easily administered orally or sublingually, and is thus an important alternative for PPH management in settings where oxytocin is not feasible.

According to the nationally representative District Level Household Survey 3 (DLHS 3), only 47% of women in India had institutional delivery in 2007–08 [3]. Therefore, a high proportion of deliveries are conducted at health sub-centres (the most peripheral and first contact point between primary health care system and the community within the Indian rural health system) or at home and are attended by traditional birth attendants or auxillary nurse midwives (ANMs). Oxytocin is often not feasable in these settings and misoprostol offers an important alternative for PPH management. Some side effects associated with misoprostol are fever, shivering, nausea, vomitting, diarrhea, abdominal pain, and chills [4–7]. Most symptoms last only for a few minutes [8]. In 2011, WHO added misoprostol to the model lists of essential medicines for preventing PPH in settings where parenteral uterotonics are not available or feasible and in 2015, misoprostol was added for treatment of PPH [9,10]. Bradley *et*. *al*. (2007) found that in low resource settings where births are attended by traditional birth attendants and where the standard care practice is to refer women with PPH to local hospitals, the use of misoprostrol is highly effective in terms of averting number of PPH cases and reducing costs [8].

The standard of care using misoprostol is to offer it as primary prevention to all mothers during delivery. Even though misoprostol has been used as primary prevention of PPH, the literature shows that 6–16% of women can still have PPH and require treatment [11–13]. Thus, a primary prevention strategy will not eliminate the need for PPH treatment. A proposed alternative stratergy is to administer misoprostol as early treatment only to those with very early signs of PPH.

This study presents per person cost of using misoprostol for primary and secondary prevention. Information on costs were collected in the context of a randomized trial in Karnataka, India, which compared primary versus secondary prevention strategies using misoprostol administered to women delivering at home or at a health sub-center.

## Materials and Methods

### The trial

The data for this study came from the trial conducted among pregnant women in Bijapur district in north Karnataka during December 2011 and March 2014 [14]. The trial was conducted by investigators from Gynuity Health Projects (New York, USA), University of Illinois at Chicago (USA), University of California, San Francisco (USA), KLE University’s Jawaharlal Nehru Medical College (Belgaum, India), and BLDE University’s Shri BM Patil Medical College (Bijapur, India).

The trial compared two community-level strategies for PPH management: primary prevention of PPH (administration of 600 mcg oral misoprostol to all women immediately after delivery) and secondary prevention of PPH (administration of 800 mcg sublingual misoprostol to women if blood loss within one hour after delivery reaches 350 ml). Historically, primary prevention was the only policy option that was being proposed and had been evaluated. Primary prevention is about preventing the onset of a health condition compared to secondary prevention which aims to detect the health condition early and then attempt to manage its impact. As such, primary prevention is preferable from an incidence point of view because it does not allow the health condition to occur thereby saving the individual from experiencing any consequences of ill health in the short or long term. However, the costs, and supply-chain burdens of primary prevention programmes are challenging [14]. The trial conducted in Karnataka was the first to propose a secondary prevention strategy of PPH and investigated the feasibility and effectiveness of the model of care in comparison to primary prevention.

The trial included deliveries conducted by Auxiliary Nurse Midwives (ANMs) at health sub-centres and women’s homes. The unit of randomization for the study was ANMs, and deliveries enrolled by each ANM constituted a cluster [14]. After considering the exclusion criteria e.g. high risk pregnancy, in active labour or refused to participate the study, there were 2,984 cases included in the cost analysis, of these 1,064 cases were in primary prevention group and 1,920 cases were in the secondary prevention group. Blood loss was measured for all women using a blood collection drape (Brasss-V Drapes, Excellent Fixable Drapes, Madurai, Tamil Nadu, India). In the primary prevention group, 1,061 (99.7 percent) women got misoprostol. In secondary prevention group, 92 (4.7 percent) women had postpartum bleeding more than 350 ml of which 90 (97.8 percent) received misoprostol.

Clinical outcomes did not differ significantly between the two arms in the trial; however, side effects were more common in the primary prevention group. Significantly more women in primary than secondary prevention experienced shivering after delivery (39.5 versus 9.0 percent, difference = -30.5, 95% CI -56.4 to -4.5) [14]. Only 4.2 and 2.2 percent of women reported moderate or severe shivering in primary and secondary groups, respectively (P = 0.151). Less than 1 percent of women in both groups described having ‘intolerable’ side effects [14].

### Data collection for cost analysis

Per person cost of misoprostol for each strategy was calculated using the ‘ingredient’ approach. All information related to training, drug use, side effects was taken from the trial data. The prices of drugs, supplies etc. were those faced by the government.

Apart from calculating the costs of the two strategies, we also calculated the cost of conducting normal deliveries at sub-centres. As there is dearth of information on the government spending on normal deliveries in sub-centres in India, we did a unit cost analysis of three randomly selected sub-centres in Bijapur district of Karnataka to find out the baseline cost–i.e. the amount government is already spending for conducting deliveries at sub-centres. We then added the additional cost for implementing the strategy–primary or secondary prevention of PPH.

### Costing methodology

The study was conducted from the government’s perspective. The Karnataka trial concludes that the secondary prevention strategy offers policymakers a feasible and practical approach to address PPH at the community level [14]. The objective of the costing study was to examine the cost difference (if any) in primary versus secondary prevention strategy of PPH using misoprostol. We chose to conduct the analysis from government perspective as along with the effectiveness of the strategy, the policymakers will also have the cost information for both strategies which will help them in decision making.

We undertook the cost analysis in two parts. The first deals with the estimation of cost of introducing misoprostol as primary or secondary prevention of PPH on a per patient basis using the trial data. In the second part, we extrapolated the cases and costs to a cohort of 14.9 million births (the number of births take place in sub-centres and home in India) [3] in each group and estimated the incremental cost to the government for both strategies.

Cost analysis of introducing misoprostol as primary or secondary prevention involved costs of training of ANMs and medical officers; cost of administering misoprostol (cost of the drug, drape and treatment of any side-effects) and cost involved in treatment of PPH.

In the second part of our analysis where we extrapolated the cases and costs to a cohort of 14.9 million births in each group, we used the proportionate number of a particular case in the sample to arrive at the number of possible cases in the population. For example, in the secondary prevention group out of 1,920 women, 90 women had blood loss of 350 ml or more after delivery. We used the fraction (90/1920 = 0.047) and multiplied by 14.9 million to get estimated number of cases in the hypothetical cohort (698,438 cases).

To calculate the incremental cost of introducing misoprostol as primary or secondary prevention of PPH, we first estimated the amount the government already spends for conducting deliveries at sub-centre. Standard costing methodology was followed to calculate the operating cost of sub-centres [15]. The average cost of normal delivery at the sub-centre was calculated using the ANM’s proportion of time spent for normal deliveries, the annual depreciated cost of capital items used for normal deliveries and the drugs and materials used for the same. ANM’s time spent for conducting deliveries and drugs and materials used for the same were collected by interviewing the ANMs during data collection from the sub-centres in July 2014. Capital cost included annual depreciated value of furniture and equipment used to conduct normal deliveries. A 3% discount rate was used [16] and country specific useful life-years for various capital items. Drugs and materials were the actuals used while conducting deliveries.

It should be noted in this context that the salaries, price of equipment used to calculate the costs of the two strategies and conducting normal deliveries were taken from the government health facilities. There could be regional variations, but we don’t expect huge variation. Further, the drugs and supplies prices used for the analysis were also taken from the approved price list of the government which is also generally standard across India.

### Ethics approval

The Institutional Review Board of the University of Illinois at Chicago, approved the protocol of the trial on 18 November 2010 and provided an updated approval on 5 September 2012. The Institutional Ethics Committee on Human Subjects Research at the Jawaharlal Nehru Medical College at KLE University, Belgaum, India, approved the protocol on 20 January 2011. The Health Ministry’s Screening Committee at the Indian Council of Medical Research, New Delhi, India, approved the protocol on 23 August 2011.

## Results

### Cost of conducting normal deliveries at sub-centres

Average cost of conducting normal deliveries at sub-centres were estimated INR 29,205 (US$487) of which 59% was human resources cost, i.e. the time cost of the ANMs for conducting deliveries. 23% of total cost was for drugs and materials used while conducting normal deliveries and rest 17% was capital cost. The cost per delivery at sub-centre was estimated INR 308 (US$5).

### Cost of strategies: Government perspective

Cost components of both the strategies using government perspective are presented in Table 1. In secondary prevention strategy as misoprostol was administered only to those having blood loss more than 350 ml, the cost of the drug is much lower. Overall, the average cost of the secondary prevention strategy is lower by INR 6 (US$ 0.1) as compared to primary prevention strategy using the government’s perspective.

**Table 1:** Cost of prevention of postpartum haemorrhage through misoprostol: Government perspective.

** Cost components**	**Number of cases**		**Total cost (INR)**	
** **	Primary Prevention	Secondary Prevention	Primary Prevention	Secondary Prevention
**Misoprostol**	1,061	90	6,918	783
**Drape**	1,064	1,920	106,400	192,000
**Drugs used for PPH cases**	1	3	14	374
Other medical supplies*	1	4	17	120
**Drugs used for controlling side effects**	155	82	527	267
**Total cost incurred**	1,064	1,920	113,876	193,197
**Average cost**			107	101
**Average training cost**			13	13
**Average cost of intervention**			120	114

*Notes*: US$1 = INR 60

*Medical supplies include IV fluid only, blood product was not considered as this is generally not freely available in government facilities in India, and this is the expenses from the patient perspective.

### Incremental cost to the government

We did the extrapolation for 14.9 million births to get enough numbers of different events for each arm. The cost per birth (either at home or at sub-centres in the cohort of 14.9 million births) was INR 308 (US$ 5.13) for both the groups. The average cost per birth after implementation of the strategy would be INR 430 (US$ 7.17) for the primary prevention group and INR 424 (US$ 7.07) for the secondary prevention group (Table 2). Therefore, the incremental cost per birth would be INR 122 (US$ 2.04) and INR 116 (US$ 1.93) for primary and secondary prevention groups respectively.

**Table 2:** Incremental cost to the government for both strategies to prevent postpartum hemorrhage (for the cohort of 14.9 million births).

**Expenditures**	**Primary Prevention**	**Secondary Prevention**
**Government already spending for conducting normal deliveries at home or sub-centres**	INR 4,589,200,000	INR 4,589,200,000
**Total cost of the strategy**	INR 6,412,677,499	INR 6,318,732,109
**Average cost per birth**	INR 430	INR 424
**Incremental cost to the government**	INR 1,823,477,499	INR 1,729,532,109
**Incremental cost per birth**	INR 122	INR 116

Note: US$1 = INR 60

## Discussion

This study estimates the cost of primary and secondary prevention strategies of preventing PPH using misoprostol. It is based on a cluster randomized trial comparing these two strategies involving around 3,000 pregnant women in Bijapur district in Karnataka, India. From a government perspective, the average cost of the primary prevention strategy is higher by INR 6 (US$ 0.1) compared to secondary prevention.

The study estimated that the government spends on average INR 308 (US$ 5.13) per birth for delivery at sub-centre or delivery assisted by skilled birth attendants (ANMs) at home. For introducing misoprostol as primary prevention of PPH (universal administration of misoprostol to women post-delivery) the government needs to spend an additional amount of INR 122 (US$ 2.04) per birth. In comparison, secondary prevention (administration of misoprostol in case blood loss ≥ 350 ml within one hour of delivery) with misoprostol would cost an additional amount of INR 116 per birth (US$ 1.93).

Implementing the primary prevention strategy costs an additional INR 6 (US$ 0.1) per birth compared to the secondary prevention strategy. In a country like India with approximately 26 million births in 2012, out of which 14.9 million births take place sub-centre and home [3], this additional cost of INR 6 per birth translates to an additional cost of INR 94 (US$ 1.6) million to the government to implement the primary prevention compared to the secondary prevention strategy. Therefore, considering secondary prevention is clinically non-inferior to primary prevention [14], implementing secondary prevention is more productively efficient. Further, the cost difference between the two strategies would increase if the price of misoprostol increases. The present calculation is based on the price of misoprostol paid by the government, if however, it needs to be purchased from open market, the price of misoprostol will increase by almost 88 percent (considering average market price of misoprostol in India). This will lead to an additional cost of INR 734 (US$12.2) million to implement primary prevention compared to secondary prevention for the cohort of 14.9 million births which take place in sub-centres or home in India.

One of the major contributors in cost differences between the two interventions is the cost of misoprostol; in a cohort of 14.9 million births, if misoprostol is given as primary prevention, the cost will be much higher than the same given as secondary prevention. The other factors such as transfers after having PPH, procedures conducted to manage PPH and stay at higher health facilities have minor influences on the cost difference and as analysis of the main trial revealed that these factors were not statistically significantly different in the two intervention arms, we have excluded those cost from the present analysis.

Another important determinant of cost is the price of the drape. Generally, drapes are not used for measuring blood loss in government health facilities in India, this was used in the trial as it was in a research mode. Therefore, the price of drape considered in this analysis is the market price. If we assume that the government will use drape for measuring blood loss, the drape price will probably be less than market price as the government will purchase in bulk. If we assume that the drape price will reduce by half i.e. from INR 100 (US$1.67) to INR 50 (US$0.83), the incremental cost per birth will reduce by INR 50 (US$0.83) in each group. Hence, there would be a huge cost saving for the government if drape is used and purchased at a cheaper rate.

As the trial showed clinical non-inferiority of secondary prevention compared to primary prevention and as there were no statistically significant differences in the clinical outcomes [14], cost-effectiveness analysis is beyond the scope of this study. Costs of these two strategies are the main area of interest.

The study has the following limitations. First, it was not possible to separate out the drugs given to manage side effects of misoprostol from the drugs which were given as routine measure after delivery for treatment of minor illnesses (e.g. antiemetic). The drug cost presented in this study may be a bit overestimated; however, it would probably be disproportionately overestimated as more side effects were reported in the primary prevention arm. Second, the laboratory test costs are actually the prices of the tests charged to the patients. We were unable to calculate the unit cost of these tests. Third, it was difficult to calculate the proportion of time that ANMs spend on delivery-related work as there is no fixed work hours of the ANMs. The sub-centres remain open as long as an ANM is available and deliveries took place even at late night. Hence, we assumed standard eight hours of work per day and six working days per week and tried to find out the proportion of time she spent conducting deliveries.

## Conclusion

The cost analysis shows that the government will have to spend an additional INR 94 (US$ 1.6) million to implement the primary prevention strategy compared to the secondary prevention strategy for controlling PPH. As clinical outcomes did not differ significantly between the two arms in the trial, taking into account the difference in costs and potential issues with sustainability, secondary prevention might be a more strategic option in a country like India where a significant number of births take place at home or sub-centres.
